# Pretreatment of Adipose Derived Stem Cells with Curcumin Facilitates Myocardial Recovery via Antiapoptosis and Angiogenesis

**DOI:** 10.1155/2015/638153

**Published:** 2015-05-05

**Authors:** Jianfeng Liu, Ping Zhu, Peng Song, Weiping Xiong, Haixu Chen, Wenhui Peng, Shuxia Wang, Shan Li, Zhiqing Fu, Yutang Wang, Haibin Wang

**Affiliations:** ^1^Department of Geriatric Cardiology, Medical School of Chinese PLA, Chinese PLA General Hospital, Beijing 100853, China; ^2^The First Surgical Department of Nanlou, Chinese PLA General Hospital, Beijing 100853, China; ^3^Department of Cardiology, Shanghai Changning Center Hospital, Shanghai 200336, China; ^4^Institute of Geriatrics, Chinese PLA General Hospital, Beijing 100853, China; ^5^Department of Cardiology, Tenth People's Hospital, Tongji University, Shanghai 200072, China; ^6^College of Life Sciences and Bioengineering, Beijing Jiaotong University, Beijing 100044, China

## Abstract

The poor survival rate of transplanted stem cells in ischemic myocardium has limited their therapeutic efficacy. Curcumin has potent antioxidant property. This study investigates whether prior curcumin treatment protects stem cells from oxidative stress injury and improves myocardial recovery following cells transplantation. Autologous Sprague-Dawley rat adipose derived mesenchymal stem cells (ADSCs) were pretreated with or without curcumin. The hydrogen peroxide/serum deprivation (H_2_O_2_/SD) medium was used to mimic the ischemic condition *in vitro*. Cytoprotective effects of curcumin on ADSCs were evaluated. Curcumin pretreatment significantly increased cell viability and VEGF secretion, and decreased cell injury and apoptosis via regulation of PTEN/Akt/p53 and HO-1 signal proteins expression. The therapeutic potential of ADSCs implantation was investigated in myocardial ischemia-reperfusion injury (IRI) model. Transplantation of curcumin pretreated ADSCs not only resulted in better heart function, higher cells retention, and smaller infarct size, but also decreased myocardial apoptosis, promoted neovascularization, and increased VEGF level in ischemic myocardium. Together, priming of ADSCs with curcumin improved tolerance to oxidative stress injury and resulted in enhancement of their therapeutic potential of ADSCs for myocardial repair. Curcumin pretreatment is a promising adjuvant strategy for stem cells transplantation in myocardial restoration.

## 1. Introduction

Myocardial infarction (MI) remains the leading cause of morbidity and mortality worldwide. The prognosis of MI is usually attributable to the extent of myocardial ischemia-reperfusion injury (IRI). The optimal treatment strategy for IRI is timely and effective reperfused ischemic myocardium. However, the reperfusion of acute ischemic myocardium can lead to myocardial stunning or even necrosis [1, 2]. The endogenous myocardial regenerative capacity is too poor to replenish a lot of dead cardiomyocytes after MI [3]. Despite advances in therapeutic intervention, including antiplatelet and antithrombotic agents and primary percutaneous coronary intervention technology, there is still no effective therapeutic method for ameliorating myocardial IRI.

Cellular cardiomyoplasty has emerged as a novel potential therapeutic modality to repair damaged myocardium. Bone marrow mesenchymal stem cells (BMSCs) have been reported to improve cardiac function and decrease fibrosis [4, 5]. It is well-known that BMSCs play the cardioprotective roles mainly through paracrine, differentiation into specialized cardiac cell lineages, and recruitment of endogenous stem cells [6, 7]. Since adipose derived mesenchymal stem cells (ADSCs) have proven to be abundant, easy to isolate and obtain repeatedly, minimally invasive properties and particularly grow old later than BMSCs, ADSCs have been an appealing cell source in regenerative medicine [8–10]. However, very low survival rate of transplanted cells is an impediment of successful heart cell therapy [11]. Although the underlying mechanisms remain undefined, the mediators of myocardial reperfusion injury, including oxidative stress, inflammation, are responsible for massive cell death [12–14]. Thus, it is imperative to enhance cell survival for cardiac regeneration in IRI myocardium.

Pharmacologically pretreatment has shown to be a rational approach in reinforcing the cells to withstand the ischemia and reperfusion injury environment [15, 16]. Curcumin is a naturally occurring yellow agent extracted from the spice turmeric that has been reported to exhibit potent antioxidant, anti-inflammatory properties [17, 18]. It has been reported that curcumin possesses the characteristic of the intracellular ROS protection and free radical-scavenging activity [19]. Curcumin can also protect against myocardial reperfusion damage by attenuation of oxidant stress [20]. Moreover, curcumin can induce heme oxygenase-1 (HO-1) expression in rat BMSCs [21]. Furthermore, recent study showed that BMSCs therapy and curcumin posttreatment synergistically improve recovery from spinal cord injury [22]. Thus, we hypothesize that pretreatment of ADSCs with curcumin could enhance antiapoptosis activity, promote angiogenesis, and improve cardiac function in myocardial IRI.

In this study, we investigated whether pretreatment of ADSCs with curcumin protected ADSCs from H_2_O_2_-induced injury, elaborating the potential antiapoptotic effect. Furthermore, to confirm the therapeutic applicability of curcumin pretreated ADSCs, we explored the role of curcumin pretreated ADSCs transplantation in promoting myocardial repair secondary to cardiac IRI injury.

## 2. Methods

### 2.1. Chemical Reagent

Curcumin was purchased from Biomol (AG-CN2-0059-M010).

### 2.2. Animals

The animal experiments were conformed to the* Guide for the Care and Use of Laboratory Animals* published by the US National Institutes of Health (NIH Publication, 8th Edition, 2011) and approved by the Institutional Animal Care and Use Committee (IACUC) of the General Hospital of Chinese People's Liberation Amy. Sprague-Dawley (SD) rats (200–250 g) were purchased from the Experimental Animal Center, the General Hospital of Chinese People's Liberation Amy. Rats were maintained under controlled conditions (12 hrs light/dark cycle) with* ad libitum* access to water and food.

### 2.3. Isolation, Expansion, and Characterization of ADSCs

Isolation and expansion of ADSCs were performed as previously described [23, 24]. Autologous ADSCs were isolated from inguinal subcutaneous adipose tissue of Sprague-Dawley male rat. Briefly, adipose tissue was rinsed with sterile phosphate-buffered saline (PBS), finely minced, and enzymatically dissociated with composite of 0.1% collagenase I (sigma) and 0.05% trypsin (sigma) in serum-free alpha-modified Eagle's medium (*α*-MEM, Gibco, USA) for 45–60 minutes at 37°C with gentle agitation. Then an equal volume of *α*-MEM/10% fetal bovine serum (FBS, Gibco) was added to neutralize enzymes. The mixture was filtered through a sterile 75 *μ*m nylon mesh to discard undigested debris. The digested tissue was centrifuged at 600 g for 5 minutes. The supernatant containing adipocytes and debris was removed. The cell pellet was resuspended in *α*-MEM supplemented with 10% FBS. The collected primary adipose tissue derived stem cells were plated in 10 cm culture plates. The dishes were maintained at 37°C, 5% CO_2_ incubator and the media were replaced every 2-3 days. The cells were monitored for confluence every day. When cells reached 80% confluence, the medium was removed and adherent cells were detached with 0.25% trypsin/0.04% EDTA (v/v 1 : 1) for 2 minutes and seeded at a ratio of 1 : 3. Cultures were passaged every 3-4 days and used for experiments from passage 3 to passage 5.

To assay the phenotype of cultured cells, surface protein markers CD34, CD31, CD45, CD90, CD105, and CD29 were assessed by fluorescence-activated cell sorting (FACS) (BD accrri C6). Briefly, cultured adherent cells isolated from adipose tissue were harvested and washed with cell staining buffer (Biolegend). Aliquots containing 1 × 10^6^ cells were incubated for 20 min at room temperature with mouse monoclonal primary antibodies to rat CD34 (Santa Cruz), CD31 (clone WM-51), CD45 (Biolgend), CD90 (Biolgend), and CD105 (Biolgend). Then the cells were washed in PBS with 2% FBS. For unlabeled antibodies, fluorescent-conjugated secondary antibodies were added at room temperature. Then cells were incubated for another 20 min. Finally, the cells were washed in cell staining buffer twice, and the samples were analyzed by FACS. Isotype-identical antibodies served as controls.

### 2.4. Viability Assay of ADSCs

The cell viability was evaluated after exposure to 100 *μ*M hydrogen peroxide (H_2_O_2_) for 12 h by trypan blue exclusion method: the proportion of viable cells = (the number of trypan blue dye negative cells/the total number of cells) × 100%.

### 2.5. Assay of LDH Release and Caspase-3 Activity

The ADSCs were pretreated with 10 *μ*M curcumin for 24 h [21]. Then, the cells were collected after being incubated with or without 100 *μ*M H_2_O_2_/serum deprivation (SD) for 12 h [23]. Cytotoxicity was evaluated in the cell supernatants through lactate dehydrogenase assay according to the manufacturer's instructions (Sigma-Aldrich, USA). To analyze caspase-3 activity, caspase-3 activity was measured in 96-well plates by using a caspase-3 activity assay kit (Cell signaling) according to the manufacturer's protocol.

### 2.6. Determination of ADSCs Apoptosis

Apoptosis was measured using an Annexin V/PI assay kit (Biolegend, San Diego, CA, USA) according to the manufacturer's instructions. The percentages of apoptotic cells were evaluated by flow cytometry (BD, Franklin Lakes, USA).

### 2.7. Western Blot

Protein samples were acquired from ADSCs and whole heart homogenates (*n* = 5/group, randomly) as reported previously [23, 24]. Proteins were isolated by sodium dodecyl sulfate-polyacrylamide gel electrophoresis (SDS-PAGE) and then transferred to a polyvinylidene difluoride (PVDF) membrane. The membranes were blocked with 5% nonfat milk for 2 h at room temperature and then incubated overnight at 4°C with antibodies against phospho-Akt (p-Akt), Akt, HO-1, cleaved-caspase-3, caspase-3 and *β*-actin (Cell Signaling Technology), VEGF, PTEN, and p53 (Santa Cruz). The membrane was subsequently washed with TBST and incubated with secondary antibodies against horseradish peroxidase-conjugated (Cell Signaling Technology). After washing with TBST, bands were detected by enhanced chemiluminescence substrate (Applygen). Staining was quantified by scanning densitometry. The experiment was repeated 5 times.

### 2.8. Enzyme-Linked Immunosorbent Assay (ELISA)

To ascertain whether curcumin pretreatment results in an increase in angiogenic humoral factor (vascular endothelial growth factor, VEGF) release from ADSCs, cells were collected after being pretreated with or without curcumin for 24 hours. The level of VEGF in culture medium supernatant was detected by VEGF ELISA kits (Sigma-Aldrich, USA) according to the manufacturer recommended protocol. All samples and standards were conducted in duplicate.

### 2.9. ADSCs Labeling, Myocardial I/R Injury Model, and ADSCs Delivery

Before autologous transplantation, the Vybrant CM-Dil cell-labeling solution (Invitrogen, USA) (50 *μ*g/mL) was added to growth media of ADSCs for 30 minutes according to the manufacturer's procedures, which can uniformly label attached culture cells.

Rats were randomly divided into the 3 groups of 24 animals each in a blind study: (1) PBS group (PBS); (2) ADSCs transplanted group (ADSCs); (3) curcumin pretreated ADSCs transplanted group (Cur-ADSCs). Autologous ADSCs were isolated from inguinal subcutaneous adipose tissue in transplanted groups before establishing IRI model.

IRI model was established by the ligation of the left coronary artery (LAD) according to a method reported previously with minor revision [25]. Rats were intraperitoneally anesthetized with pentobarbital (65 mg/kg). The tracheal incubation and ventilator assisted ventilation had been performed. Electrocardiogram was maintained to monitor ischemia and arrhythmia during the ischemia/reperfusion surgery. After the left 4/5th intercostals chest region was opened, the LAD was identified and gently ligated with a 6.0 prolene suture. Successful ischemia was assessed by visual myocardial blanching and the widening of QRS complex and typical ST segment elevation in electrocardiograph. The ligature was removed after 60 min and reperfusion ascertained visually. Finally, the opened thorax was closed. During the surgical procedures, the depth of anaesthesia was monitored by using absence of the pedal withdrawal reflex, slow constant breathing, and no response to surgical manipulation. Buprenorphine was intraperitoneally administered before and after the surgery (0.05 mg/kg). None of rats died at the end of the experiment.

One week after LAD ligation, rats chest was reopened and total of 4 × 10^6^ ADSCs in 100 *μ*L PBS were separately injected into three different foci in the ADSCs transplanted group (33.3 *μ*L/site). Two administration sites were in the peri-infarct regions and one was within the infarct area. 100 *μ*L PBS alone was administered to PBS-treated rats.

### 2.10. Echocardiography

Serial echocardiographic measurements were assessed in all rats prior to surgery at baseline and 28 days after cell transplantation. Animals (*n* = 6/group) were anaesthetized using 1.5–2.0% isoflurane, followed by function assessment with 14.0 MHz Ultrasound imaging System (Acuson, Germany). Both two-dimensional images and M-mode interrogation were obtained to evaluate left ventricular systolic and diastolic function. The left-ventricular end-diastolic diameter (LVEDD) and left-ventricular end-systolic diameter (LVESD) were measured. LV fractional shortening (FS) and LV ejection fraction (EF) were calculated as follows: FS(%) = [(LVEDD − LVESD)/LVEDD] × 100; EF(%) = [(EDD^3^ − ESD^3^)/EDD^3^] × 100. The images captured at the end of cardiac systole and diastole were used to measure LV end-systolic volume (ESV) and end-diastolic volume (EDV). All parameters measurement was averaged based on at least 3 consecutive cardiac cycles.

### 2.11. Histology

The rats were euthanized after 7 days of transplantation (*n* = 18/group) for cell retention, 2,3,5-triphenyltetrazolium chloride (TTC) staining, and TUNEL assay. At the end of the experiments (*n* = 6/Group), immunohistochemistry was used to assess microvessel density. After being injected an overdose of sodium pentobarbital in compliance with above guidelines, hearts were quickly removed, fixed in 4% paraformaldehyde, embedded into O.C.T compound, quickly frozen in −80°C, and then processed for cryosectioning (4 *μ*m thickness). Ten sections were prepared at 10 different transversal levels at the site of tissue necrosis, equally distributed from base to apex.

To analyze the retention of ADSCs in ischemic myocardium (*n* = 6/Group), the sections were stained with an anti-cTnI antibody. Cell retention was investigated by identification of Dil staining expression under fluorescent microscopy. The numbers of Dil^+^ cells and DAPI in each slide were calculated. The data were exhibited as the percentage of Dil^+^/DAPI.

### 2.12. *In Situ* Myocardial Apoptosis Assay

The immunofluorescent measurement of apoptotic cells was examined as reported previously [24, 26]. Briefly, myocardial frozen sections were analyzed using terminal deoxynucleotidyl transferase dUTP nick end labeling (TUNEL) staining according to the manufacturer's instructions (MEBSTAIN Apoptosis kit II; Takara). The color images were captured randomly at high magnification and digitized by using a camera connected to an Olympus microscope. For each heart sample, 50 high resolution fields (10 sections at different transversal levels, 5 fields for each section) were chosen and counted in a blinded manner. The cells with clear nuclear labeling were characterized as TUNEL positive cells.

### 2.13. Assessment of Infarct Size

Cardiac infarct size was measured by TTC staining method as reported previously [27]. Briefly, TTC staining was used to evaluate cardiac viability and to confirm the infarct size. The tissue sections were incubated in a 1% TTC (Sigma) solution (pH 7.4) for 20 minutes at 37°C. The sections were fixed in 10% PBS formalin solution for overnight at 2–8°C. Viable tissue stained by TTC showed a deep red color, and TTC-negative pale area is considered as infarct area. The size of MI was calculated as a percentage of the sectional region of the infarct tissue of the left ventricle to the sectional area of the whole left ventricle. The images of each TTC-stained tissue slice were captured by a digital camera.

### 2.14. Measurement of Capillary Density

Capillary density was analyzed in the slice stained with anti-vWF antibody (1 : 200, Sigma). Cryosections were fixed with acetone for 30 minutes and endogenous peroxidase was quenched with 3% H_2_O_2_. After blocking with 2% normal goat serum, slices were incubated with the anti-vWF antibody at 4°C overnight. Then, FITC-conjugated IgG were incubated for 2 hours at room temperature before imaging under laser confocal microscope (FV1000, Olympus). The number of capillaries was calculated in five randomly chosen high magnification fields.

### 2.15. Statistical Analysis

Data are expressed as mean ± SD. Statistics were analyzed with 17.0 SPSS software. Statistical difference between two groups was determined by Student's *t*-test. Results for more than two groups were estimated by one-way ANOVA with least significant difference test. *P* < 0.05 was regarded as significant difference.

## 3. Results

### 3.1. Phenotypes of Isolated ADSCs

**Figure 1:**
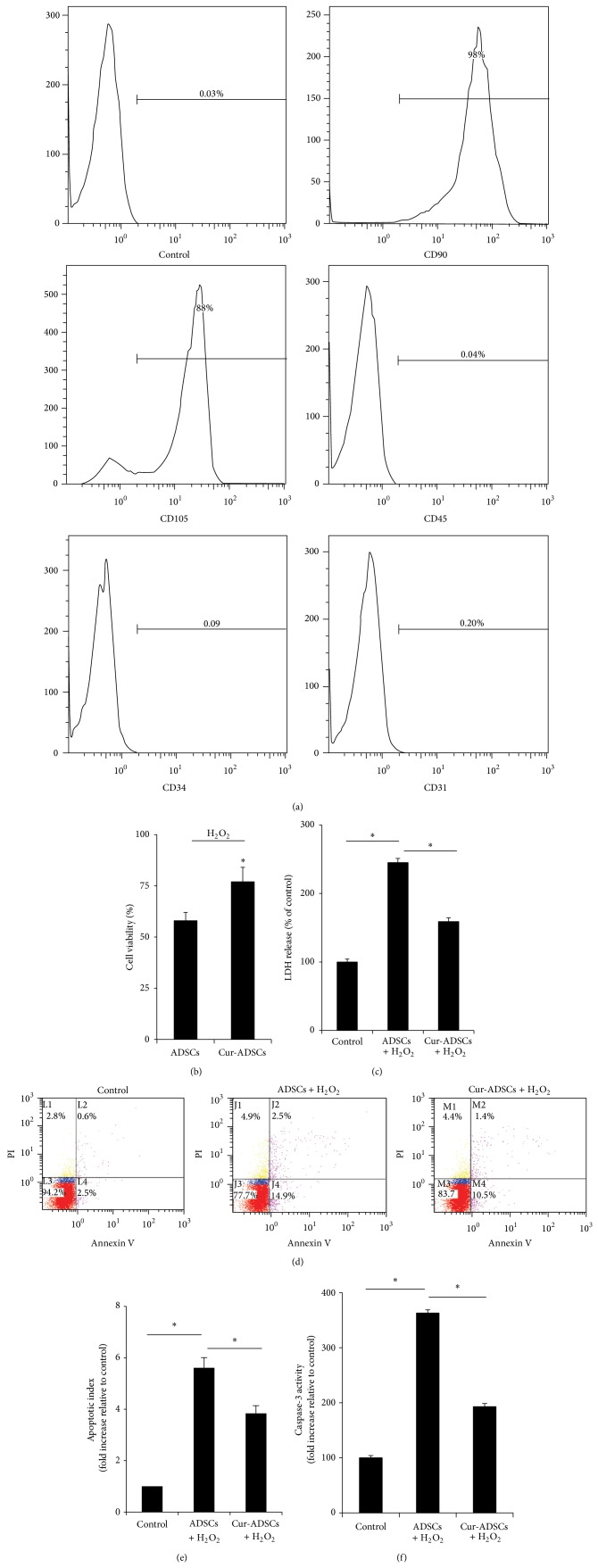
Immunophenotype characterization of ADSCs and cytoprotective effects of curcumin pretreatment on ADSCs. (a) Expressions of cell phenotypes. CD90 and CD105 are positive. CD31, CD34, and CD45 are negative. (b) Trypan blue exclusion analysis showed cell viability was improved by curcumin pretreatment. (c) Quantitative analysis of LDH production in the cell supernatant revealed curcumin pretreatment reduced cells injury. ((d) and (e)) Representative Annexin V/PI staining showed curcumin pretreatment prevented apoptosis of ADSCs (*P* < 0.05). (f) Curcumin pretreatment protected ADSCs against H_2_O_2_/SD induced caspase-3 activation (*P* < 0.05). ^∗^ *P* < 0.05.

The surface proteins of adherent cultured ADSCs were analyzed by flow cytometry. After the three passages, most of these ADSCs expressed CD90 and CD105. In contrast, little expression of the hematopoietic and endothelial markers, including CD31, CD34, and CD45, was observed (Figure 1(a)).

### 3.2. Cytoprotective Effects of Curcumin Pretreatment on ADSCs

The effects of pretreatment with curcumin on the viability, cytotoxicity, and injury of ADSCs were evaluated. The trypan blue measurement showed better survival of ADSCs in the curcumin pretreatment group compared with the ADSCs alone group (Figure 1(b); *P* < 0.05). Similarly, curcumin pretreated ADSCs were significant protected against H_2_O_2_, as evidenced by reduction in LDH leakage as compared with the control group (Figure 1(c); *P* < 0.05). The curcumin pretreated ADSCs showed lower apoptosis than H_2_O_2_-treated ADSCs (Figures 1(d) and 1(e); *P* < 0.05). Likewise, caspase-3 activity was reduced in ADSCs subjected to H_2_O_2_, while pretreatment of ADSCs with curcumin indicated a marked enhancement in cellular caspase-3 activity (Figure 1(f); *P* < 0.05).

### 3.3. Curcumin Pretreatment Regulates PTEN/Akt/p53 and HO-1 Signaling Proteins and Enhances Paracrine Factor Release

**Figure 2:**
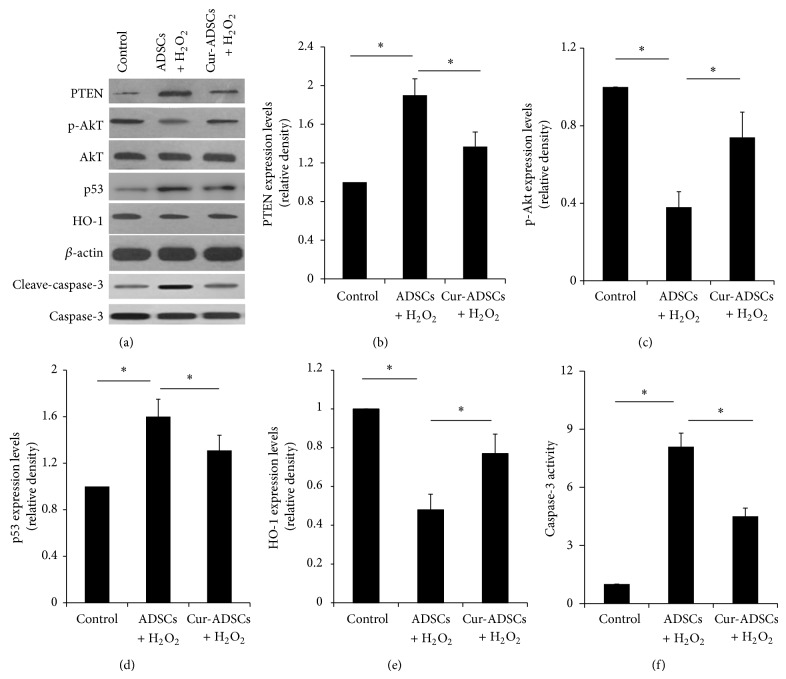
Effects of curcumin pretreatment on PTEN/Akt/p53 and HO-1 signaling proteins expression. (a) Representative Western blots of PTEN, p-Akt, Akt, p53, HO-1, cleave-caspase-3, caspase-3, and *β*-actin. ((b)–(f)) Quantitative analysis of PTEN, p-Akt, Akt, p53, HO-1, and cleave-caspase-3. The results showed that curcumin pretreatment significantly increased the expression of p-Akt and HO-1 and reduced the PTEN, p53, and cleave-caspase-3 expression (*P* < 0.05). ^∗^ *P* < 0.05.

**Figure 3:**
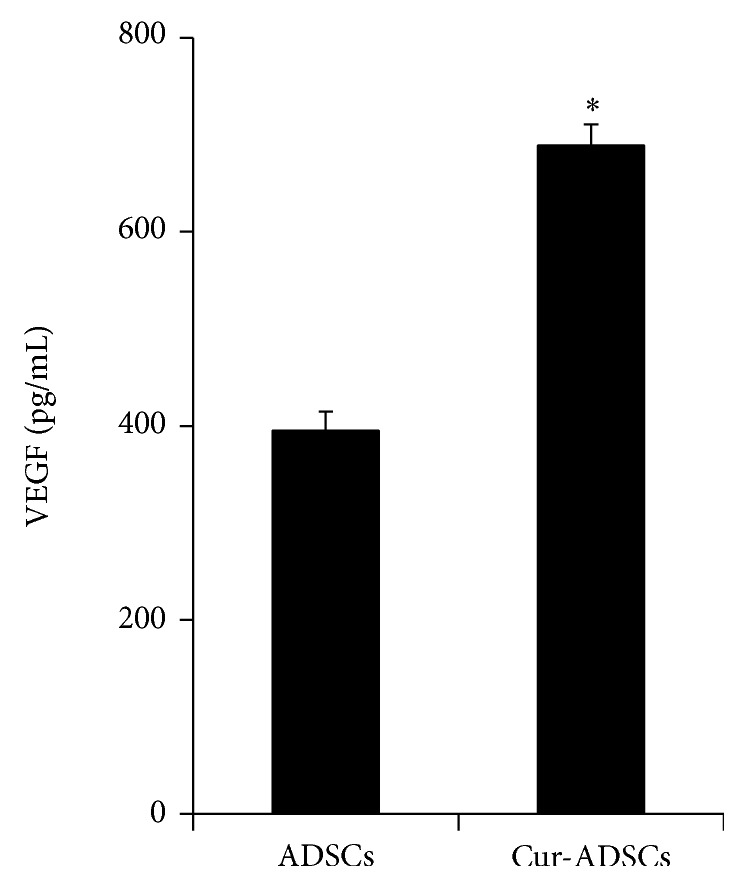
Effect of curcumin pretreatment on paracrine effect. A significant production of VEGF from ADSCs pretreated by curcumin was observed when compared with nontreated ADSCs control (*P* < 0.05). ^∗^ *P* < 0.05 versus ADSCs.

The changes in prosurvival and proapoptotic molecules expression of several candidate proteins were evaluated after with or without H_2_O_2_ by Western blotting. Although the expression of survival-related p-Akt and HO-1 markedly decreased in ADSCs group compared to in control group, their levels were rescued in curcumin pretreatment group (Figures 2(a), 2(c), and 2(e); *P* < 0.05). As Akt signaling molecules are associated to the proapoptotic protein, such as PTEN, p53, and caspase-3, we found that H_2_O_2_ treatment significantly increased the PTEN, p53, and caspase-3 expression in ADSCs, which was reversed partly by curcumin pretreatment (Figures 2(a), 2(b), 2(d), and 2(f); *P* < 0.05). In addition, the VEGF production of ADSCs increased after pretreatment with curcumin compared to the control group (Figure 3; *P* < 0.05).

### 3.4. Injection of Curcumin Pretreated ADSCs Improves Cardiac Function

**Figure 4:**
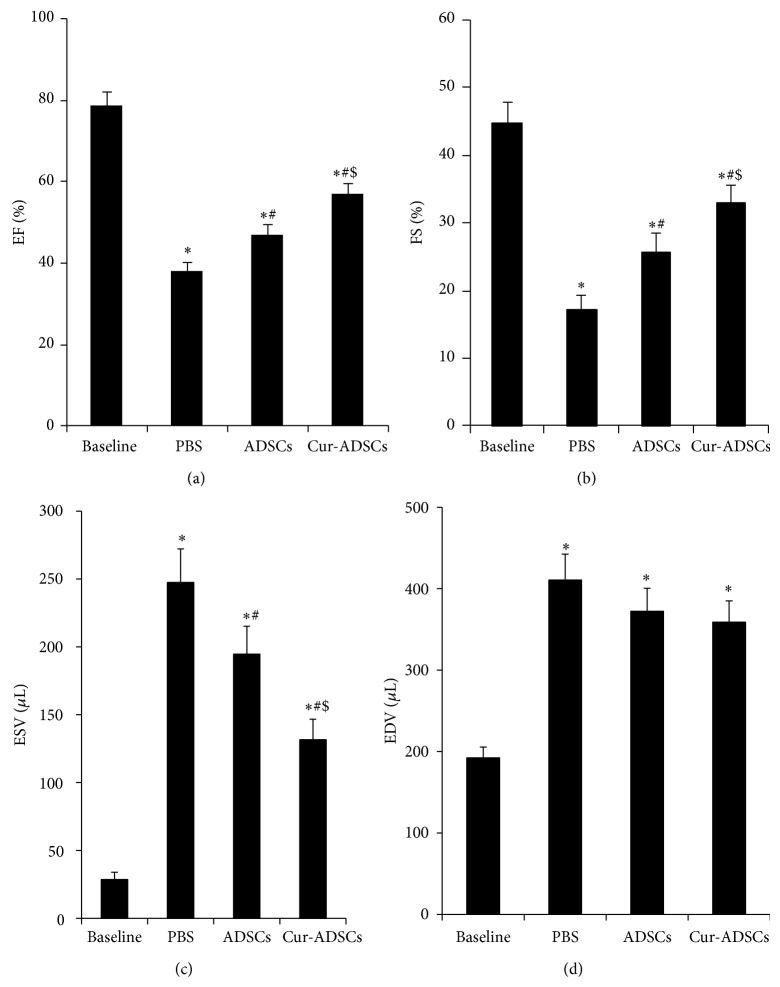
Echocardiographic assessment of cardiac function. ((a)-(b)) EF and FS were highest in the Cur-ADSCs group compared with PBS control and ADSCs groups (*P* < 0.05). (c) ESV was significantly improved in the Cur-ADSCs group compared with PBS control and ADSCs groups (*P* < 0.05). (d) There is no significant difference for EDV among PBS, ADSCs, and Cur-ADSCs groups. Statistical differences (*P* < 0.05) are indicated from the baseline (∗), PBS (#), and ADSCs ($).

Cardiac function was assessed by echocardiogram at baseline and day 28 after transplantation. We observed a significant decrease in the EF and FS at day 28 after transplantation, which demonstrated that the rats MI model was performed successfully. EF and FS were significantly increased in transplantation of ADSCs and Cur-ADSCs groups compared with PBS group. Moreover, the best EF and FS were obtained for hearts with transplanted Cur-ADSCs (Figures 4(a) and 4(b); *P* < 0.05). Additionally, ESVs were markedly reduced in ADSCs and Cur-ADSCs groups compared to the PBS group (Figure 4(c); *P* < 0.05). However, the LV EDVs showed no significant difference among the 3 groups (Figure 4(d); *P* < 0.05). These results unambiguously demonstrated that curcumin pretreated ADSCs transplantation had a beneficial effect on cardiac function in MI hearts.

### 3.5. Curcumin Pretreatment Facilitates the Survival and Retention of Transplanted ADSCs

**Figure 5:**
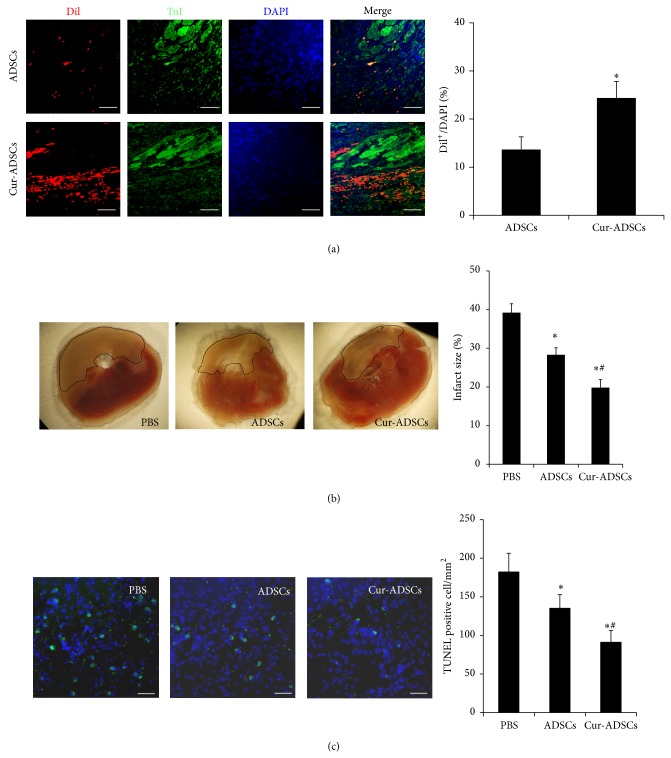
Administration of pretreated ADSCs increases cells retention and decreases myocardial fibrosis and apoptosis after myocardial infarction at 7 days after transplantation. (a) Representative fluorescence microscopic images ADSCs (Dil^+^, red florescence), cardiomyocytes (cTnI, green florescence), and DAPI (blue fluorescence) at 1 week after injection. Quantitative analysis revealed that a higher retention of ADSCs in Cur-ADSCs group was observed. Scale bars = 50 *μ*m. ^∗^ *P* < 0.05 versus ADSCs. (b) Effect of ADSCs transplantation and/or pretreatment with curcumin on infarct size. Representative TTC staining images and quantitative analysis at 7 days after transplantation showed intramyocardial injection of ADSCs decreased the LV infarct size (*P* < 0.05). Scale bars = 100 *μ*m. Statistical differences (*P* < 0.05) are indicated from PBS (∗) and ADSCs (#). (c) Measurement of apoptosis in the ischemic heart. Representative TUNEL staining images in the peri-infarct area of heart slices by immunofluorescence and quantitative analysis showed TUNEL positive cells were the least in the Cur-ADSCs group (*P* < 0.05). Scale bars = 100 *μ*m. Statistical differences (*P* < 0.05) are indicated from PBS (∗) and ADSCs (#).

The survival and retention of ADSCs were confirmed by calculating the ratio of Dil^+^/DAPI at 1 week after injection. The Dil^+^ cells could be noticed in the Cur-ADSCs group (Figure 5(a)). The ratio of Dil^+^/DAPI in Cur-ADSCs group significantly increased compared to that in the ADSCs alone group (24.3 ± 3.5% versus 13.6 ± 2.7%; *P* < 0.05). The result showed that curcumin could markedly facilitate the retention and survival of transplanted cells in infarct myocardium.

### 3.6. Curcumin Pretreated ADSCs Implantation Reduces Fibrotic Area

7 days after transplantation, a significant decrease in left ventricle infarct area was observed in ADSCs group compared with the PBS treated control group (28.3 ± 1.8% versus 39.2 ± 2.3%, *P* < 0.05) (Figure 5(b)). Furthermore, infarct size was notably smaller in Cur-ADSCs group compared to that in the PBS group (19.8 ± 2.0% versus 39.2 ± 2.3%, *P* < 0.05) (Figure 5(b)). These results revealed that curcumin pretreatment has the protective effects on myocardial remodeling.

### 3.7. Curcumin Pretreated ADSCs Significantly Inhibit Myocardial Apoptosis

The incidence of apoptotic cells in the peri-infarct area of PBS treated rats was 182.3 ± 24.1/mm^2^, which was significantly decreased to 135.2 ± 17.6/mm^2^ in ADSCs group (Figure 5(c); *P* < 0.05). Rats implanted with Cur-ADSCs exhibited even further reduction in the percentage of TUNEL positive cells (91.2 ± 15.1/mm^2^; *P* < 0.05) (Figure 5(c)).

### 3.8. Curcumin Pretreatment Increases Angiogenesis and VEGF Expression Level in Postischemic Myocardium

**Figure 6:**
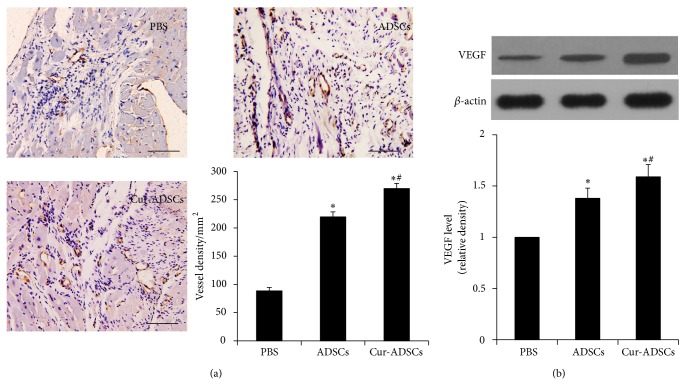
Assessment of vessel density at the border zone of MI and VEGF level in ischemic myocardium. (a) Representative anti-vWF antibody staining images by immunohistochemistry and quantitative analysis revealed microvessel density was the highest in the Cur-ADSCs group (*P* < 0.05). (b) Representative Western blots of VEGF expression level and quantitative analysis showed curcumin pretreatment significantly VEGF level in ischemic myocardium. *β*-actin is used as internal parameter. Scale bars = 100 *μ*m. Statistical differences (*P* < 0.05) are indicated from PBS (∗) and ADSCs (#).

Immunohistochemical tissue sections showed that implantation of ADSCs significantly enhanced vascular density when compared to PBS treated group (*P* < 0.05, Figure 6(a)). The microvessel density of Cur-ADSCs treated hearts was further improved compared to that of rats receiving ADSCs injection (*P* < 0.05, Figure 6(a)). The quantitative results suggested that curcumin pretreatment could enhance the neovascularization in response to ADSCs transplantation.

Western blotting revealed that a significant upregulation in VEGF level was observed in ADSCs group and Cur-ADSCs group compared to that in PBS group (*P* < 0.05, Figure 6(b)). Notably, highest rise was seen in Cur-ADSCs group (*P* < 0.05, Figure 6(b)). The results indicated that curcumin pretreatment enhances angiogenesis in ischemic myocardium which is related to VEGF signal.

## 4. Discussion

The present study demonstrated the major findings were as follows. (1) Curcumin pretreatment markedly enhanced ADSCs antiapoptotic ability and preserved their viability* in vitro*, in part, by inhibition of PTEN, p53 and, caspase-3 signaling proteins and activation of Akt and HO-1 signaling proteins, which possessed proapoptosis and prosurvival function, respectively. (2) Curcumin pretreatment augmented ADSCs production of VEGF, which contributed to neovessels formation and improving cells survival. (3) Curcumin pretreatment improved cardiac function, enhanced cells engraftment, reduced infarct size, and promoted neovascularization in peri-infarct area.

One of the major challenges in cells transplantation therapy is the problem of poor survival in the harsh microenvironment of the ischemic myocardium. One mechanism which may be responsible for massive loss of donor ADSCs is the serious oxidative stress conditions in ischemic milieu. In view of the fact that ROS had been reported to adversely influence stem cell functional properties and result in detachment-induced apoptosis [28, 29], it is imperative to attenuate the extent of injected cells apoptosis in infarcted heart. Pretreatment is a promising protective approach with stem cell engraftment [11]. In this study, we utilized H_2_O_2_ to mimic ROS microenvironment and found that ADSCs can be pretreated by curcumin to reduce their apoptosis and enhance viability* in vitro*. Previous study showed that curcumin can attenuate H_2_O_2_-induced endothelial cell oxidative stress injury. It is also reported that curcumin can protect myocardium from reperfusion injury by attenuation of oxidant stress and mitochondrial dysfunction [20]. Thus, cellular oxidative stress, which can influence ADSCs survival and viability, could be ameliorated in part by curcumin.

The PTEN/Akt pathway had been proven to play a pivotal role in regulating cell apoptosis and angiogenesis [30, 31]. It is widely known that PTEN remains the major negative regulator of Akt signaling [32]. Recent studies had reported that phosphoinositide 3-kinase [PI3K]/Akt and HO-1 are associated with the regulation of stem cell fate [21, 33]. For example, the PI3K/Akt pathway has been shown to be involved in maintaining embryonic stem cell (ESC) pluripotency [34]. Additionally, p53 is implicated in protecting cells from oxidative stress insult [35]. Particularly, curcumin was reported to modulate PTEN/AKT/p53 axis to exhibit its protective effects in MCF-7 breast cancer cells [36]. And curcumin was also found to increase HO-1 expression of rat bone marrow-MSCs [21]. In the present study, our data demonstrated that the cytoprotective effects of curcumin pretreatment were dependent on part phosphorylation of Akt, increasing expression of HO-1, inhibition of PTEN, p53, and caspase-3 expression. Moreover, the variation tendency of caspase-3, as a downstream proapoptotic protein, is opposite to that of Akt and HO-1. These results lay the foundation for enhancement of cells retention and survival, which is confirmed* in vivo* experiment.

Another major effect of our experiments* in vitro* was that curcumin pretreatment can significantly promote VEGF secretion in ADSCs. In parallel to the improved paracrine result, the data* in vivo* showed that capillary densities were markedly enhanced in the curcumin pretreated cell group. It is well-known that VEGF, as a potent angiogenic factor, plays an important role in regulation of angiogenesis and that it also is implicated in the initiation of new blood vessel formation [37]. In addition, our data suggested that curcumin pretreated cell transplantation obviously decreased the myocardial apoptosis. That is similar to the report that VEGF can inhibit various cellular apoptosis and improve their survival [38]. We also found that ADSCs pretreated with curcumin significantly improved cardiac function and decreased myocardial fibrosis in the infarct heart. Similarly, VEGF was reported to exert a significantly negative effect on ischemic cardiac remodeling [39]. Thus, it is possible that curcumin pretreatment may play an important part in apoptotic ADSCs and ischemic myocardium through antioxidant activity and paracrine antiapoptotic cytokine, VEGF.

In this study, ADSC therapy led to an increase in LV function and a reduction in end-systolic volume but did not significantly affect end-diastolic volume, suggesting that the primary mechanism of effect with ADSC delivery may be at the level of acute injury and improved contractility, versus chronic LV remodeling. Since more precise assessments of chronic remodeling were not performed, including cardiac and cardiomyocyte hypertrophy and remote region fibrosis, it is difficult to assess whether ADSC therapy directly impacted remodeling. The reduction in infarct size at 7d after transplant may be associated with an early reduction in scar expansion and/or secondary cell death.

## 5. Conclusion

The present study demonstrated that curcumin effectively protects the ADSCs from oxidative stress via regulation of PTEN/Akt/p53 and HO-1 signal proteins and promotes the VEGF release from ADSCs, which facilitated enhancement of cardiac function, improvement of cells retention, modification of the ischemic microenvironment, and decrease of fibrosis in MI hearts. Autologous ADSCs therapy adjuvant with curcumin pretreatment is a promising therapeutic strategy for myocardial restoration.
